# Prevalence of diarrheagenic *Escherichia coli* virulence genes in the feces of slaughtered cattle, chickens, and pigs in Burkina Faso

**DOI:** 10.1002/mbo3.30

**Published:** 2012-07-22

**Authors:** Assèta Kagambèga, Outi Martikainen, Anja Siitonen, Alfred S Traoré, Nicolas Barro, Kaisa Haukka

**Affiliations:** 1 Bacteriology Unit, Department of Infectious Disease Surveillance and Control, National Institute for Health and Welfare (THL)P.O. Box 30, FI-00271, Helsinki, Finland; 2 Laboratoire de Biologie Moléculaire, d'Epidémiologie et de Surveillance bactéries et virus transmis par les aliments; CRSBAN, Département de Biochimie-Microbiologie, UFR-SVT/Université de Ouagadougou03 B.P. 7021, Ouagadougou 03, Burkina Faso

**Keywords:** Cattle, chickens, diarrheagenic *Escherichia coli*, multiplex PCR, pigs, virulence genes

## Abstract

We investigated the prevalence of the virulence genes specific for five major pathogroups of diarrheagenic *Escherichia coli* (DEC) in primary cultures from feces of animals slaughtered for human consumption in Burkina Faso. For the study, 704 feces samples were collected from cattle (*n* = 304), chickens (*n* = 350), and pigs (*n* = 50) during carcass processing. The presence of the virulence-associated genes in the mixed bacterial cultures was assessed using 16-plex polymerase chain reaction (PCR). Virulence genes indicating presence of DEC were detected in 48% of the cattle, 48% of the chicken, and 68% of the pig feces samples. Virulence genes specific for different DECs were detected in the following percentages of the cattle, chicken, and pig feces samples: Shiga toxin-producing *E. coli* (STEC) in 37%, 6%, and 30%; enteropathogenic *E. coli* (EPEC) in 8%, 37%, and 32%; enterotoxigenic *E. coli* (ETEC) in 4%, 5%, and 18%; and enteroaggregative *E. coli* (EAEC) in 7%, 6%, and 32%. Enteroinvasive *E. coli* (EIEC) virulence genes were detected in 1% of chicken feces samples only. The study was the first of its kind in Burkina Faso and revealed the common occurrence of the diarrheal virulence genes in feces of food animals. This indicates that food animals are reservoirs of DEC that may contaminate meat because of the defective slaughter and storage conditions and pose a health risk to the consumers in Burkina Faso.

## Introduction

Animals carry harmless *Escherichia coli* in the intestines as part of the normal gut flora. Sometimes, they are carriers of pathogenic *E. coli* strains that can cause gastrointestinal illness in humans. The importance of these diarrheagenic *E. coli* (DEC) in causing foodborne diseases has been understood in recent years in Africa (Okeke 2009), but very little is known about the reservoirs and routes of the infection on the continent. In general, meat products are considered to be an important source of DEC infections. The meat can be contaminated due to the poor hygiene practices during slaughter. Therefore, adherence to good hygienic practices in slaughter and meat production are essential for prevention of microbial carcass contamination and for ensuring the meat quality and health protection (FAO 2005). Healthy asymptomatic animals may carry zoonotic pathogens and represent a reservoir for DEC, which may enter the food chain via the weak points in hygiene practices of the slaughter process (Hussein 2007; Islam et al. 2008; Rhoades et al. 2009). The animals also play an important role in fecal contamination of drinking water sources and agricultural crops enabling direct transfer of zoonotic organisms to humans (Blanco et al. 2003).

At least five main pathogroups of *E. coli* have been associated with human acute intestinal infection. They can be classified based on their virulence genes: Shiga toxin-producing *E. coli* (STEC), enteropathogenic *E. coli* (EPEC), enterotoxigenic *E. coli* (ETEC), enteroaggregative *E. coli* (EAEC), and enteroinvasive *E. coli* (EIEC) (Nataro and Kaper 1998). STEC produces Shiga toxins encoded by s*tx1* and/or s*tx2* or their variants (Table 1). Besides the *stx* gene(s), STEC strains often carry the *eae* gene, encoding the adherence factor intimin. Also, they often express additional virulence factors, such as enterohemolysin (Schmidt et al. 1995). STEC can cause gastroenteritis that may be complicated by hemorrhagic colitis or hemolytic–uremic syndrome (HUS), which is the main cause of acute renal failure in children (Paton and Paton 1998).

**Table 1:** The *Escherichia coli* pathogroups and their marker genes targeted in the study

Pathogroup	Gene	Locus	Virulence mechanism	References
STEC	*stx1*	Phage	Shiga toxin 1	Paton and Paton 1998
STEC	*stx2*	Phage	Shiga toxin 2	Paton and Paton 1998
STEC	EHEC*-hly*	Virulence plasmid pO157	Enterohemolysin	Paton and Paton 1998
STEC, EPEC	*eae*	LEE pathogenicity island in the chromosome	Intimin, a protein causing attaching / effacing lesions	Nataro and Kaper 1998
STEC, EPEC	*escV*	LEE pathogenicity island in the chromosome	A conserved area in LEE pathogenicity island	Müller et al. 2007
STEC, EPEC	*ent*	OI-122 pathogenicity island in the chromosome	Enterotoxin or enterohemolysin, a homolog to ShET2 enterotoxin of *Shigella flexnerii*	Müller et al. 2007; Afset et al. 2008
Typical EPEC	*bfpB*	EPEC adherence factor (EAF) plasmid	Subunit of bundle forming pilus (BFP)	Nataro and Kaper 1998; Müller et al. 2007
ETEC	*elt*	Plasmid	Heat-labile enterotoxin LT-I	Nataro and Kaper 1998
ETEC	*estIa*	Plasmid or transposon	Heat-stable enterotoxin ST-Ip	Nataro and Kaper 1998
ETEC	*estIb*	Plasmid or transposon	Heat-stable enterotoxin ST-II	Nataro and Kaper 1998
EIEC	*invE*	Virulence plasmid pINV	Transcription regulator, regulates the *ipa* genes	Hale 1991; Müller et al. 2007
EIEC	*ipaH*	Virulence plasmid pINV and the chromosome	Invasion plasmid antigen	Hale 1991; Hsu et al. 2010
EAEC	*aggR*	Chromosomal island, plasmid pAA	AggR regulon, transcription activator, regulates the genes of fimbrial biogenesis	Cerna et al. 2003; Harrington et al. 2006
EAEC	*pic*	Chromosome	Serine protease	Henderson et al. 1999; Müller et al. 2007
STEC, EPEC, ETEC, EIEC, EAEC	*astA*	Plasmid	EAEC heat-stable enterotoxin (EAST-1)	Nataro and Kaper 1998
STEC, EPEC, ETEC, EIEC, EAEC	*uidA*	Chromosome	*β*-Glucuronidase	Blanco et al. 1982; Müller et al. 2007

EPEC produces characteristic histopathology known as attaching and effacing (A/E) on intestinal cells (Schmidt 2010) (Table 1). EPEC is further divided into two subtypes, typical (tEPEC) and atypical (aEPEC), depending on the presence or absence of the EPEC adherence factor (EAF) plasmid (Nataro and Kaper 1998; Schmidt 2010). Strains of aEPEC occur frequently also in developed countries, whereas tEPEC is the leading cause of infantile diarrhea in developing countries (Trabulsi et al. 2002). ETEC produces heat-labile (LT) and/or heat-stable (ST) enterotoxins and is an important cause of diarrhea in infants and travelers (Kaper et al. 2004). EIEC is associated with invasive, bloody diarrhea resembling that caused by *Shigella* sp. Invasion is mediated by the genes encoding, for example, Ipa proteins and their transcription regulator *invE* (Nataro and Kaper 1998; Lan et al. 2004). EAEC harbors the mechanism for aggregative-adherence pattern mediated by aggregative-adhesive fimbriae. It is increasingly recognized as a diarrheal pathogen in developing countries (Huang et al. 2004).

In our previous study on the retail meats sold at the markets of Ouagadougou, 44% of the beef and 29% of the chicken samples were found to contain DEC virulence genes (Kagambèga et al. 2012). We hypothesized that DEC bacteria might originate especially from fecal contamination of meat during slaughter. In developing countries such as Burkina Faso, the carcass-processing and meat-selling conditions are defective (Kagambèga et al. 2011), which can lead into further proliferation of the pathogens. Recent information is available on the occurrence of DEC in animals in sub-Saharan Africa, such as in Uganda (Majalija et al. 2008), in Ethiopia (Mersha et al. 2010), in Nigeria (Ojo et al. 2010; Akanbi et al. 2011), but little is known about the situation in Burkina Faso. To estimate the risks and the appropriate measures to avoid the risks related to the hygiene of the slaughter process, it is necessary to collect data related to the animals that are shedding potential zoonotic pathogens. This article describes the prevalence of DEC virulence genes in fresh feces collected from slaughtered cattle, chickens, and pigs in Ouagadougou.

## Materials and Methods

### Sampling

From March to August 2010, we collected altogether 704 fecal samples from cattle (*n* = 304) and pigs (*n* = 50) after slaughter at the central abattoir, and from chickens (*n* = 350) from the local poultry meat sellers in Ouagadougou, Burkina Faso. There were no records available concerning the origin of the animals, but according to the abattoir or the poultry sellers, animals were received from different areas across the country. Immediately after the animals were slaughtered, the rectal material was collected aseptically. The samples were transported to the laboratory and kept at 4°C until the microbiological examination was started within 8 h. Subsequently, of each fecal sample, 25 g was homogenized in 225 mL buffered peptone water (Liofilchem, Teramo, Italy) and enriched at 37°C for 24 h. The enriched samples were cultured on Sorbitol MacConkey (SMAC) agar (Oxoid, Basingstoke, England) at 37°C overnight. Bacterial mass from each plate was collected and stored at −30°C in tubes containing 1 mL of brain heart infusion broth with 15% (v/v) glycerol for further analysis.

### 16-plex polymerase chain reaction (PCR)

The presence of DEC virulence genes in the feces samples was detected using 16-plex PCR targeting the genes *uidA, pic, bfpB, invE*, EHEC*-hlyA, elt, ent, escV, eaeA, ipaH, aggR, stx1, stx2, estIa, estIb*, and *astA* after recultivation the bacterial mass on Cystine Lactose Electrolyte Deficient (CLED) agar (Difco, Sparks, USA). The primers, sample preparation, and PCR conditions were as previously described (Antikainen et al. 2009; Kagambèga et al. 2012). The following genes were considered indicative of the *E. coli* pathogroups: for STEC, the presence of *stx1* and/or *stx2* and possibly *eaeA*, *escV*, *ent*, and EHEC-*hly*; for EPEC, the presence of *eaeA* and possibly *escV*, *ent*, and *bfpB*, the absence of *bfpB* indicated atypical EPEC; for ETEC, the presence of *elt* and/or *estIa* and/or *estIb*; for EAEC, the presence of *pic* and/or *aggR*; for EIEC, the presence of *invE* and *ipaH*. The gene *uidA* was used as a general marker for *E. coli*. As *astA* was not specific for a certain pathogroup, it was not utilized in the final analysis.

The following reference strains were used: RH4283 (E 2348/69; Baldini et al. 1983) for EPEC, RH3533 (ATCC 35401) for ETEC, RH4270 (ATCC 43895) for STEC, RH6647 (145-46-215, Statens Serum Institute [SSI], Copenhagen, Denmark) for EIEC, and IH56822 (patient isolate; Keskimäki et al. 2000) for EAEC. The negative controls were *E. coli* RHE6715 (ATCC25922) and sterile distilled water. All the 16-plex PCR positive results were confirmed by single PCRs.

### Statistical analysis

The chi-square test or Fisher's exact test of OpenEpi version 2.3.1 were used to determine the statistical significance of the data.

## Results

The 16-plex PCR was used to detect the selected virulence genes carried by five pathogroups of *E. coli*. Virulence genes of at least one DEC pathogroup was detected in 348 (49%) of the 704 feces samples, with 149 (21%) being positive for virulence genes of STEC, 172 (24%) of EPEC, 40 (6%) of ETEC, 58 (8%) of EAEC, and 5 (1%) of EIEC (Table 2). Among the different animals, STEC virulence genes were more prevalent in cattle (37%) and pigs (30%) than in chickens (6%). The lower prevalence of STEC virulence genes in chickens than in cattle and pigs was statistically significant (*P* < 0.001). Chickens and pigs had a higher prevalence of EPEC virulence genes (37% and 32%, respectively) than cattle (8%) (*P* < 0.001). ETEC virulence genes were found in 12 (4%), 19 (5%), 9 (18%) and EAEC virulence genes in 22 (7%), 20 (6%), and 16 (32%) of cattle, chicken, and pig feces samples, respectively. The higher prevalences of ETEC and EAEC virulence genes in pigs than in cattle or chickens were statistically significant (*P* < 0.001 for both). EIEC virulence genes were found in 5 (1%) of the chicken feces samples. Finding of virulence genes specific for several pathogroups of DEC in samples was common. Virulence genes of more than one pathogroup were detected in 64 (9%) of all the studied samples, in 23 (8%) of the cattle feces samples, in 26 (7%) of the chickens feces samples, and in 15 (30%) of the pig feces samples. However, determination of the copresence of STEC and EPEC virulence genes was not possible using a PCR method, because STEC strains may contain all the virulence genes present in atypical EPEC.

**Table 2:** Diarrheagenic *Escherichia coli* (DEC) pathogroups present in the animal feces, based on the detection of their virulence genes by PCR

	Number of DEC, *n* (%)			
	
DEC pathogroups	Cattle (*n* = 304)	Chickens (*n* = 350)	Pigs (*n* = 50)	Total (*n* = 704)
Any DEC	145 (48)	169 (48)	34 (68)	348 (49)
STEC	112 (37)	22 (6)	15 (30)	149 (21)
EPEC	25 (8)	131 (37)	16 (32)	172 (24)
ETEC	12 (4)	19 (5)	9 (18)	40 (6)
EAEC	22 (7)	20 (6)	16 (32)	58 (8)
EIEC	0	5 (1)	0	5 (1)

A DEC-positive sample may contain virulence genes of several pathogroups.

Table 3 shows the occurrence of the 14 genes, which were utilized to analyze the PCR results. Among the 149 samples, which based on the PCR detection were positive for STEC virulence genes, *stx1* without *stx2* was detected in 46 (31%) samples, *stx2* without *stx1* in 36 (24%) samples, and both *stx1* and *stx2* in 67 (45%) samples. Shiga toxin genes together with the genes indicating the presence of LEE pathogenicity island, that is, *eaeA* and/or *escV*, were detected in 53 (36%) of the STEC virulence gene-positive samples. EHEC-*hly* was detected in 34 (23%) of the STEC virulence gene-positive samples and in 3 (2%) of the EPEC virulence gene-positive samples. None of the EPEC virulence gene-positive samples had *bfpB*, so all of them appeared to contain atypical EPEC. Among the 40 ETEC virulence gene positive samples, *estIb* without *estIa* or *elt* was most common with 17 (43%) positive samples, followed by *elt* alone with 10 (25%) positive samples, and *estIa* alone with 6 (15%) positive samples. Five EIEC virulence gene positive samples had *invE*, but no *ipaH*.

**Table 3:** Number of virulence genes detected by 16-plex PCR in 304 cattle, 350 chicken, and 50 pig feces samples and in the six control strains

		Virulence genes													
		
Pathogroups		*stx1*	*stx2*	*hly*	*eaeA*	*escV*	*ent*	*bfpB*	*elt*	*estIa*	*estIb*	*aggR*	*pic*	*ipaH*	*invE*
	**Control strains**														
STEC	RH4270	+	+	+	+	+	+	−	−	−	−	−	−	−	−
EPEC	RH4283	−	−	−	+	+	+	−	−	−	−	−	−	−	−
ETEC	RH3533	−	−	−	−	−	−	−	+	−	+	−	−	−	−
EAEC	IH56822	−	−	−	−	−	−	−	−	−	−	+	+	−	−
EIEC	RH6647	−	−	−	−	−	−	−	−	−	−	−	−	+	+
Negative control	RH6715	−	−	−	−	−	−	−	−	−	−	−	−	−	−
	Cattle*														
STEC	93	73	68	18	10	14	5	−	−	−	−	−	−	−	−
EPEC	22	−	−	1	11	21	11	−	−	−	−	−	−	−	−
EAEC	7	−	−	−	−	−	−	−	−	−	−	4	4	−	−
STEC + ETEC	7	5	6	2	2	1	−	−	2	1	5	−	−	−	−
STEC + EAEC	9	6	7	2	2	3	1	−	−	−	−	6	3	−	−
EPEC + ETEC	1	−	−	−	1	1	−	−	−	1	−	−	−	−	−
EPEC + EAEC	2	−	−	−	−	2	1	−	−	−	−	2	−	−	−
ETEC + EAEC	1	−	−	−	−	−	−	−	1	−	−	−	1	−	−
STEC + EAEC + ETEC	3	3	3	−	2	−	1	−	−	−	3	2	2	−	−
	Chickens*														
STEC	15	10	7	3	3	10	2	−	−	−	−	−	−	−	−
EPEC	113	−	−	1	66	108	31	−	−	−	−	−	−	−	−
ETEC	6	−	−	−	−	−	−	−	4	1	2	−	−	−	−
EAEC	4	−	−	−	−	−	−	−	−	−	−	2	2	−	−
EIEC	5	−	−	−	−	−	−	−	−	−	−	−	−	5	−
STEC + ETEC	4	4	−	1	−	3	−	−	−	1	3	−	−	−	−
STEC + EAEC	3	1	2	−	1	1	1	−	−	−	−	3	−	−	−
EPEC + ETEC	6	−	−	−	2	5	2	−	−	2	4	−	−	−	−
EPEC + EAEC	10	−	−	−	4	10	3	−	−	−	−	9	1	−	−
ETEC + EAEC	1	−	−	−	−	−	−	−	1	−	−	−	1	−	−
EPEC + EAEC + ETEC	2	−	−	−	2	2	1	−	−	1	1	1	1	−	−
	Pigs*														
STEC	9	6	7	5	−	5	1	−	−	−	−	−	−	−	−
EPEC	7	−	−	−	6	7	2	−	−	−	−	−	−	−	−
EAEC	3	−	−	−	−	−	−	−	−	−	−	2	1	−	−
STEC + ETEC	2	2	1	2	1	2	−	−	1	−	1	−	−	−	−
STEC + EAEC	2	2	1	1	1	1	−	−	−	−	−	2	−	−	−
EPEC + EAEC	4	−	−	1	3	4	1	−	−	−	−	4	−	−	−
STEC + EAEC + ETEC	2	1	1	−	−	2	−	−	2	−	1	2	−	−	−
EPEC + EAEC + ETEC	5	−	−	−	5	5	−	−	4	2	2	5	−	−	−

* Number of the positive cattle/chicken/pig samples for each pathogroup or pathogroup combination.

## Discussion

This study is the first to be undertaken in Burkina Faso to investigate the occurrence of virulence genes specific for DEC in slaughtered animals. The results suggested that animals used for meat production are commonly carriers of the main diarrheagenic pathogroups of *E. coli*. Especially, both STEC and EPEC virulence genes were detected in about a quarter of the samples, whereas ETEC, EAEC, and EIEC virulence genes were less frequently detected.

Of the animals slaughtered for human consumption, 37% of the cattle, 6% of the chicken, and 30% of the pig feces were positive for STEC virulence genes. Also, previously, the ruminants have been indicated as the main natural reservoir for human STEC infections (Caprioli et al. 2005; Cookson et al. 2006; Gyles 2007; Hussein 2007). Investigations on the prevalence of STEC are most commonly based on detection of the O157 serogroup; its detected prevalence in cattle feces varies widely from 0% to over 50% (Rhoades et al. 2009). Based on detection of the *stx* genes, as many as 73% of the healthy cattle in Bangladesh (Islam et al. 2008), 70% in France (Pradel et al. 2000), 69% in Japan (Kobayashi et al. 2001), 25% in Australia (Fagan et al. 1999), and 19% in India (Das et al. 2005) were found to be STEC positive. In Africa, in Nigeria, 10% of cattle were found to be positive for the selected seven STEC serogroups (Ojo et al. 2010).

Analysis of the STEC virulence genes revealed that bovine feces in Burkina Faso mostly harbor both *stx*1 and *stx2*, followed by *stx1* alone or *stx2* alone. Similar observations have been made in India (Das et al. 2005) and Germany (Strockbine et al. 1998; Schmidt et al. 1999). In contrast, abundance of *stx2* alone was common in France (Rogerie et al. 2001), Japan (Kobayashi et al. 2001), and Argentina (Blanco et al. 2004). Based on the PCR analysis, it is not possible to know whether the genes detected originate from one or several STEC strains harbored in an animal.

In our study, pigs were found to carry STEC virulence genes nearly as frequently as cattle. It is possible that the freely roaming pigs in Burkina Faso get infected through cattle feces and then serve as an additional possible reservoir for human infections. In Australia, 21% of pig feces tested using PCR were detected to carry *stx* genes (Sidjabat-Tambunan and Bensink 1997). In general, pigs are not considered as major carriers of human pathogenic STEC, although some STEC O157 strains from pigs have been isolated (Heuvelink et al. 1999; Johnsen et al. 2001; Bonardi et al. 2003; Caprioli et al. 2005). However, F18 fimbriae-positive *E. coli* producing the Shiga toxin variant Stx2e are significant pathogens of pigs (da Silva et al. 2001).

In chickens, we detected 6% prevalence of STEC virulence genes. This is comparable with 9% of STEC found from chicken feces in Tanzania (Raji et al. 2006). However, in several other studies no STEC was detected in poultry (Heuvelink et al. 1999; Kobayashi et al. 2002) or the prevalence was low compared with that in cattle (Schouten et al. 2005; Dipineto et al. 2006).

We found EPEC virulence genes to be more prevalent in chicken (37%) and pig (32%) feces than in cattle feces (8%). These numbers are probably an underestimate, as several samples might actually have contained EPEC in addition to STEC. It was not possible to detect their coinfection by a direct PCR technique. However, the 37% prevalence of EPEC virulence genes in chicken feces was in line with the previous reports where retail chickens were found to be commonly contaminated by EPEC (Lee et al. 2009); we found 29% prevalence in Burkina Faso (Kagambèga et al. 2012). In Argentina, up to 26% of cloacal swabs of chicken and 58% of the slaughtered chicken carcasses were positive for EPEC (Alonso et al. 2011). The 8% prevalence of EPEC virulence genes in cattle feces in this study was lower than 31% found in cattle feces in New Zealand (Cookson et al. 2006). The prevalence of EPEC virulence genes in pigs (32%) was higher than 18% found in pigs in Germany (Krause et al. 2005). Only virulence genes of atypical EPEC were detected in this study. Typical EPEC is, indeed, rarely isolated from animals, whereas atypical EPEC strains are isolated from both animals and humans (Nataro and Kaper 1998; Aktan et al. 2004; Krause et al. 2005). In our previous study among children in Burkina Faso, atypical EPEC were detected in the feces of diarrheagenic children more often than typical EPEC (Bonkoungou et al., in press).

ETEC and EAEC virulence genes were found at lower rates than STEC and EPEC virulence genes from the feces of the studied animals with an exception of 32% prevalence of EAEC virulence genes in pigs. In a previous study in Brazil (Uber et al. 2006), the EAEC isolates from calves, piglets, and horses were found to differ genetically from the human isolates. The strains of animal origin lacked *aggR*, but harbored other marker genes, such as *pic*. Among the feces samples in this study, *aggR* was more common than *pic*. Furthermore, in our previous study, we found *aggR* from raw meat samples, but never *pic* (Kagambèga et al. 2012).

ETEC is a major cause of severe diarrheal disease in suckling and weanling animals. For the pig industry, ETEC diarrhea is causing considerable losses also in Africa. In Zimbabwe, 32% of the studied piglets tested positive for STa, STb, LT, or Stx-2e genes (Madoroba et al. 2009). Animal-derived strains are known to produce enterotoxins similar to those of human strains, but colonization factors necessary for colonization of the host small bowel are species specific and different in human and animal strains (Qadri et al. 2005). In our study, *estIb* was found to be most prevalent among the 40 ETEC virulence gene-positive samples followed by samples positive for *elt* and *estIa*. In our previous study on raw meat samples, we detected different proportions of these genes, *elt* being the most common and *estIb* the least common (Kagambèga et al. 2012).

EIEC virulence genes were detected only in chicken feces in this study. However, detection of the EIEC-specific genes *ipaH* and *invE* can also indicate the presence of *Shigella* sp. in the sample. For both EIEC and *Shigella*, the reservoir is considered to be the gut of infected humans (Meng et al. 2007). Finding of this pathogroup in chicken feces might be explained by the typical close contact of the chickens and humans in developing countries, where chicken roam freely on the yards and the surroundings.

The role of pigs as a reservoir for diarrheal DEC has not been commonly recognized. However, in this study, we found rather high occurrence of STEC, EPEC, and EAEC virulence genes in pig feces. Furthermore, virulence genes specific for more than one DEC pathogroup were mostly found among the pigs. Therefore, pigs may be a noteworthy reservoir for several DEC pathogroups in Burkina Faso. However, comparison of isolates from both animal and human sources would be needed to evaluate the zoonotic risk for humans. Yet, in future studies, more attention should be given to pigs as a potential source of zoonotic DEC infections, because in developing countries they often live in close contact with humans. The results from the previous studies, where the similarity of the *E. coli* isolates from humans and animals has been investigated are somewhat ambiguous (Cookson et al. 2010; Clermont et al. 2011). In the studies conducted in Africa, Kariuki et al. (1999) concluded that although several different pulsed-field gel electrophoresis genotypes of *E. coli* were isolated from children and chickens from the same farms in Kenya, the *E. coli* strains from these two sources were distinct. Rwego et al. (2008) ended up in quite the opposite conclusion in Uganda, suggesting that both rates of human–livestock interactions and patterns of human hygiene affect human–livestock bacterial transmission in the rural setting they studied.

The high prevalence of DEC virulence genes detected in this study suggests widespread occurrence of DEC in the feces of slaughtered cattle, chickens, and pigs in Burkina Faso. This finding together with the defective meat retail conditions creates a potential infection route for foodborne pathogens. Good hygienic practices at slaughterhouses and processing plants as well as at home are necessary to minimize the risk of human DEC infections. The multiplex PCR approach is well suited for rapid and sensitive detection of the presence of the DEC virulence genes.
